# 

**Published:** 1895-03

**Authors:** 


### CONTENTS.

ORIGINAL ARTICLES—

Cholecystotomy, in one and two sittings. By J. McF. Gaston, M. D., Atlanta, Ga......... ..................................... 123

A Case of Peritoneal Adhesions of Head of Colon Simulating Appendicitis. By A. F. Harrington, A. B., M. D., Atlanta, Ga. 128

Treatment of Compound Fractures of Skull, with report of case. By William S. Goldsmith, M. D., Atlanta. Ga........... 130

The Use of Pepto Mangan for Anæmia in Pulmonary Tuberculosis. By Karl Von Ruck, B. S., M. D., Asheville, N. C....... 135

The Coal Tar Derivatives By J. W. Duncan, Atlanta, Ga...... 140

SELECTIONS AND ABSTRACTS—

A Case of Strangulated Femoral Hernia During Pregnancy.... 129
Abnormality of Genital Organs.... 134
Woman on the Wheel.... 139
A Gem Dedicated to that Rabelias of the Profession.... 143
Thyroidectomy in the Treatment of Goitre.... 144
Repeated Washings of the Stomach in Opium Poisoning.... 154

SOCIETY REPORTS—

Academy of Medicine and Surgery, Richmond, Va.... 155
Maryland Clinical Society.... 165

EDITORIAL—

Medical Association of Georgia.... 171
Special Notes.... 172
Prescriptions.... 174

